# Unlocking the potential of high-dimensional quantum communication with scalable photonic entanglement in time and frequency

**DOI:** 10.1126/sciadv.aee1333

**Published:** 2026-07-01

**Authors:** Kai-Chi Chang, Murat Can Sarihan, Nicky Kai Hong Li, Florian Kanitschar, Kemal Enes Akyuz, Yujie Chen, Dong-Il Lee, Jin Ho Kang, Alwaleed Aldhafeeri, Andrew Mueller, Matthew D. Shaw, Boris Korzh, Maria Spiropulu, Paul Erker, Marcus Huber, Chee Wei Wong

**Affiliations:** ^1^ Fang Lu Mesoscopic Optics and Quantum Electronics Laboratory, Department of Electrical and Computer Engineering, University of California, Los Angeles, CA 90095, USA.; ^2^ Atominstitut, Technische Universität Wien, Stadionallee 2, 1020 Wien, Austria.; ^3^ Institute for Quantum Optics and Quantum Information, Austrian Academy of Sciences, Boltzmanngasse 3, 1090 Wien, Austria.; ^4^ Center for Digital Safety and Security, AIT–Austrian Institute of Technology, Giefinggasse 4, 1210 Wien, Austria.; ^5^ Jet Propulsion Laboratory, California Institute of Technology, 4800 Oak Grove Dr., Pasadena, CA 91109, USA.; ^6^ Applied Physics, California Institute of Technology, 1200 E California Blvd., Pasadena, CA 91125, USA.; ^7^ Department of Applied Physics, University of Geneva, 1205 Geneva, Switzerland.; ^8^ Division of Physics, Mathematics and Astronomy, California Institute of Technology, 1200 E California Blvd., Pasadena, CA 91125, USA.

## Abstract

High-dimensional photonic entanglement holds substantial promise for advancing quantum communication, computation, and metrology. For example, large-alphabet quantum communication protocols are known to benefit from enhanced noise resilience and information capacity via multibit time-bin encoding. Yet, characterizing high-dimensional entangled states is challenging, as full-state tomography becomes prohibitively costly and often requires unrealizable measurements. Here, we demonstrate a scan-free method to characterize high-dimensional entanglement in the time-frequency domain. Our reconstruction achieves a record 5.70 ± 0.07 ebits and a fidelity of 65.4 ± 0.4% with the maximally entangled state of local dimension 1021, certifying the presence of 668-dimensional entanglement. We further prove the attainability of a secure key rate of 15.6 kilobits per second in a composable finite-size, entanglement-based protocol and show that in continuous operation, the setup can quickly approach asymptotic key rates. Using commercial telecom components and state-of-the-art low-jitter single-photon detectors, our scalable architecture offers a practical path toward high-rate, noise-resilient quantum communication test beds.

## Teaser

A time-frequency entanglement experiment enables record 5.7 ebits and paves the way for scalable high-dimensional QKD.

## INTRODUCTION

Quantum photonic qudits are a crucial resource for high-dimensional quantum information processing (*1*–*4*), environment-resilient quantum key distribution (QKD) (*5*, *6*), superdense coding (*7*–*10*), quantum computation (*11*–*15*), and quantum imaging (*16*, *17*). The availability of large Hilbert space dimensionalities within the photonic degrees of freedom (DoFs)—such as frequency bins (*18*–*22*), time bins (*23*–*32*), temporal modes (*33*–*35*), orbital angular momentum (*36*–*43*), path (*11*, *44*, *45*), and pixel bases (*46*–*48*)—enables the encoding of vast amounts of information with fewer photons compared to qubit-based protocols that rely solely on the polarization DoF. However, certifying experimentally generated high-dimensional entangled states is a crucial and challenging task for entanglement in any DoF (*3*, *4*). Specifically, the high dimensionality of these states, such as those produced by the generation of photon pairs, presents an intriguing challenge regarding their measurement (*3*, *4*). The number of projective measurements required for full-state tomography (FST) scales quadratically with the dimensionality of the Hilbert space being examined. To tackle this issue, several quantum tomographic methods have been introduced and experimentally demonstrated, such as adaptive tomographic approaches (*49*, *50*), compressed learning (*51*), mutually unbiased bases (MUBs) in the spatial domain (*39*, *46*, *48*, *52*), machine learning (*53*), and interferometric methods (*26*, *29*, *54*). However, these techniques are either constrained by a priori hypotheses on the quantum state under study (*26*, *29*, *49*–*51*) or by the limited speed and efficiency of the data acquisition (*39*, *46*, *48*, *53*, *54*) in the certification of the high-dimensional quantum states. For large-alphabet QKD, for example, although proof-of-principle entanglement-based qudit QKD has been examined (*55*–*59*), the security relies on many assumptions and is thus not comparable with contemporary qubit implementations while showing promising signs of potentially high key rates (*57*, *59*). Here, we address the secure key rate challenge in the specific context of temporally and spectrally correlated biphoton states. We focus on the particular challenge of reconstructing relevant features of the two-photon coincidence postselected quantum state emerging from spontaneous parametric down-conversion (SPDC), specifically in the temporal basis. These quantum states exhibit strong correlations in time and frequency (*4*), observed within the plane of biphoton generation, a characteristic also seen in other photon-pair sources based on spontaneous four-wave mixing (*1*, *2*).

The prevalent method in the literature for reconstructing the quantum state emitted by a nonlinear medium relies on local projective techniques (*25*, *27*–*31*, *39*, *46*–*48*); this approach suffers from drawbacks related to the measurement times, as it requires successive measurements on non-orthogonal bases, and especially in the spatial domain, every outcome is associated with either a different filter setting or another detector, rendering the scaling to high dimensions prohibitively slow or expensive. Here, we introduce a scan-free approach that addresses both issues, offering complete reconstruction of the joint temporal intensity (JTI) of the biphoton state. This information can be visualized by discretizing the arrival time of the biphoton state, defined as the marginals of the coincidence distribution obtained by integrating over the coordinates of one of the biphotons. Then, we can reconstruct the intrinsic JTI of SPDC from postprocessing the single measurement. The other measurement is a frequency-resolved JTI from the time-to-frequency converter: Here, such a converter is realized in the commercially available ±10,000 ps/nm dispersion emulator and compensator modules with optical loss less than 3 dB. We demonstrate a notable capability, where the straightforward dual-basis measurements allow the retrieval of the JTI of the biphoton states in arbitrary temporal modes. In our scheme, the measurement time typically takes only a few seconds depending on the source brightness, losses in telecom fiber components, and the detection efficiency of single-photon detectors. In contrast, previous projective techniques might require several hours of measurement, even with a smaller subset of modes.

With our proposed approach, we first certify the high-dimensional entanglement, both in terms of distillable entanglement and entanglement dimensionality. By using time-bin encoding and fiber-optic telecom components, in conjunction with our low-jitter single-photon detectors, our results successfully witness up to 5.70 ± 0.07 ebits and 668-dimensional entanglement, both of which are records, considering prior high-dimensional quantum photonic qudit systems (*26*, *29*, *37*, *39*, *46*–*48*). Our technique also presents markedly faster measurements (three orders of magnitude faster for 61 × 61 dimensions and six orders of magnitude faster for 1021-dimensions compared to prior works using the fidelity bound method (*39*, *46*–*48*), with reliable characterization of biphoton quantum states. For the key throughput challenge, we develop a scalable semidefinite programming–based method capable of certifying composable security against coherent and collective attacks from finite sample sizes. This work thus represents a key step toward realizing a fully scalable high-dimensional quantum photonic platform using the energy-time DoF for a high-rate quantum communication fiber link.

## RESULTS

### High-dimensional photonic qudits in time-frequency from SPDC

**Fig. 1.**
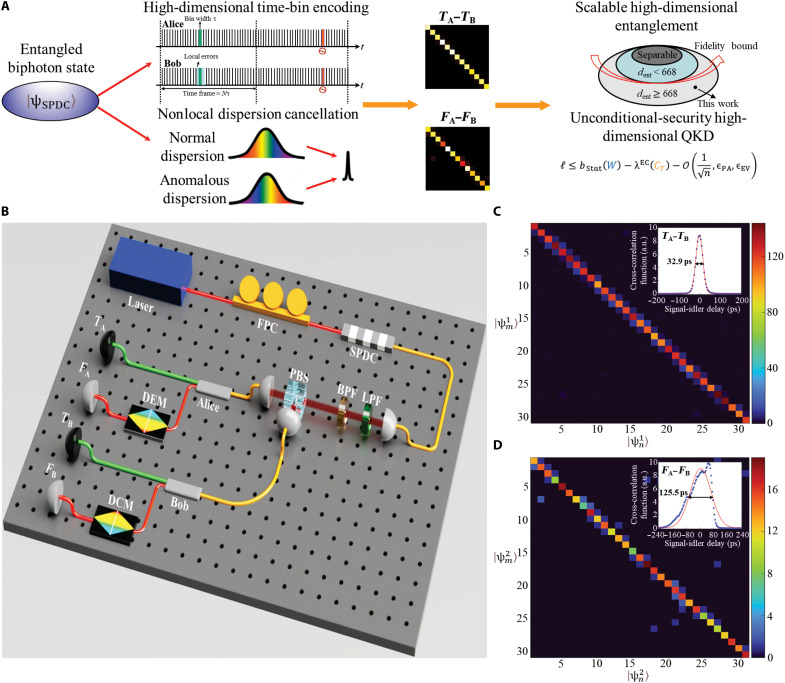
Two-shots measurements for high-dimensional qudit entanglement, high-rate QKD, and 31-dimensional time-frequency–resolved JTIs. (**A**) In SPDC photons, the detection of one photon fixes the arrival time of the other photon, yielding strong temporal correlations in the JTI. We denote the temporal measurement basis of Alice and Bob as $T_{A}-T_{B}$. By using the time-to-frequency convertor, we can perform the frequency-resolved measurements in basis $F_{A}-F_{B}$. For $T_{A}-T_{B}$, the local timing jitter errors are marked as light blue slots, while the bin width τ and the number of bins *N* define the time frame length *N*τ, optimized for the JTI. Orange slots indicate that there are no registered coincidence photons. For $F_{A}-F_{B}$, we use nonlocal dispersion cancellation to recover the narrow temporal correlation and to convert temporal information of SPDC into the frequency domain. (**B**) The experimental setup involves separating signal and idler photons, with Alice and Bob each using 50:50 fiber beam splitters and SNSPDs for both $T_{A}-T_{B}$ and $F_{A}-F_{B}$ measurements. (**C** and **D**) Exemplary 31-dimensional JTIs in $T_{A}-T_{B}$ and $F_{A}-F_{B}$ bases. The FWHMs of the temporal correlation peak are 32.9 and 125.5 ps, respectively. For (D), we optimize the $F_{A}-F_{B}$ measurements by adjusting the pump wavelength. The slight asymmetry of the temporal correlation peak comes from the limitation of the time-to-frequency convertor. Parameters (τ, *N*) are chosen to optimize the JTI: τ = 200 ps, *N* = 1024 for $T_{A}-T_{B}$; τ = 800 ps, *N* = 1024 for $F_{A}-F_{B}$. The duration of coincidence counting for the data in (C) and (D) is 3 s, and no accidental subtraction is used. a.u., arbitrary units.

The high-dimensional Hilbert space is a discretization of the intrinsic continuous time and frequency correlation of SPDC, where a three-wave mixing process generates the signal and idler photons (*1*, *2*, *4*). Such strong quantum correlations are typically characterized by the JTI and joint spectral intensity measurements (*4*, *29*, *30*, *35*). Figure 1A describes the two-shot measurements, enabled by the arrival-time encoding and the time-to-frequency converter. For the first measurement, we assigned the temporal measurement basis to be $T_{A}-T_{B}$, where *T*_A_ and *T*_B_ are the measurement bases corresponding to the arrival times at Alice and Bob, respectively. For the second measurement, we use a nonlocal dispersion cancellation technique to retrieve the narrow temporal correlation of biphoton state and map the temporal measurements into frequency-resolved measurements (*59*), termed $F_{A}-F_{B}$. In both measurements, the JTI of SPDC photons can be measured by discretizing the arrival times of the biphoton state. This process involves high-dimensional temporal encoding, and we define the marginals of the coincidence distribution by integrating over the coordinates of one of the biphotons. Local timing jitter errors are light blue slots, and there are two key parameters to optimize the JTI: bin width τ and the number of bins *N*. The time frame length is hence defined as the product of bin width τ and the number of bins *N*. Orange slots indicate that there are no registered coincidence photons. We note that the JTI measurements reconstructed by this study are dimensionally independent from the large-alphabet encoding nature of arrival times. The JTI measurements are only limited by the detectable coincidence counts from the experimental quantum photonic platforms. With this approach, we can reconstruct the JTIs of SPDC by postprocessing the two-shot measurements: $T_{A}-T_{B}$ and $F_{A}-F_{B}$ form the two approximate measurement MUBs for evaluating high-dimensional entanglement witnesses and proving security parameters for QKD.

### Experimental setup and measured MUBs

Building on the principle detailed in the previous section, we experimentally developed a platform using large-alphabet time-bin encoding and the time-to-frequency converter to reconstruct the biphoton state. This biphoton quantum state is emitted via SPDC in a type II process, where the energy-time entangled photon pairs generated from our continuous laser–pumped nonlinear $\chi^{\left(2\right)}$ waveguide are expressed in the time domain as

$$\varphi_{\text{biphoton}}\propto \int {\mathrm{dt}}_{-}\varphi_{\text{biphoton}}\left(t_{-}\right){|t_{+}+t_{-}\rangle }_{\text{Signal}}⊗{|t_{+}-t_{-}\rangle }_{\text{Idler}}$$ (1)

where $t_{+}=\left(t_{\text{Signal}}+t_{\text{Idler}}\right)/2$, and $t_{-}=\left(t_{\text{Signal}}-t_{\text{Idler}}\right)/2$. $\varphi_{\text{biphoton}}\left(t_{-}\right)$ is the joint temporal amplitude, and its magnitude square ${|\varphi_{\text{biphoton}}\left(t_{-}\right)|}^{2}$ is the JTI of $t_{-}$. This JTI of biphoton is known to be difficult to measure in energy-time DoF, often due to the limitation of detection jitter (*1*, *2*, *4*).

A visual representation of the experimental setup for the high-dimensional arrival-time encoding to discretize the JTI is shown in Fig. 1B [see Materials and Methods for more details]. With the SPDC-generated photon pairs, the continuous wave filtered, with the entangled signal and idler photons separated by a polarization beam splitter. Both Alice and Bob use their 50:50 fiber beamsplitters for conducting biphoton temporal correlation measurements ($T_{A}-T_{B}$) and frequency-resolved correlation measurements ($F_{A}-F_{B}$). Each side uses two low-jitter superconducting nanowire single-photon detectors (SNSPDs) for detection *(**60**,* *61**)*. The inset in Fig. 1C depicts the measured cross-correlation of biphotons in a temporal basis ($T_{A}-T_{B}$) using two SNSPDs with low jitter. In this temporal measurement basis, the second-order correlation peak has a full width at half maximum (FWHM) measured at ≈32.9 ps, constrained by the detector and electronic jitter within our coincidence counting module. For the frequency-resolved measurement bases *F*_A_ and *F*_B_, we insert a pair of time-to-frequency converters of ±10,000 ps/nm dispersion emulator and compensator modules (DEM and DCM, respectively), with the optical loss less than 3 dB. Via nonlocal dispersion cancellation (*58*, *59*), we retrieve the narrow correlated temporal peak with an FWHM of about 125.5 ps, bounded by the detectors and the dispersion modules we used, as shown in the inset of Fig. 1D. The effective frequency resolution in this measurement is obtained as FWHM timing jitter normalized by the applied dispersion, corresponding to ≈0.00329 nm (0.41 GHz), sizably smaller than our SPDC source FWHM bandwidth of ≈250 GHz. For our measurements in Fig. 1D, we optimize the frequency-resolved *F*_A_ and *F*_B_ measurements by adjusting the pump wavelength, and the slight asymmetry of the temporal correlation peak comes from the imperfection of our dispersive components. With both the temporal and frequency correlated bases, subsequently, we can capture the arrival-time stamps of coincidences originating from these two-shot measurements. Figure 1 (C and D) shows an example of the resulting discretized 31-dimensional large-alphabet JTIs in both $T_{A}-T_{B}$ and $F_{A}-F_{B}$ measurement bases. For the $T_{A}-T_{B}$ basis in Fig. 1C, we choose a 200-ps bin width τ and the number of bins *N* at 1024; for the *F*_A_ and *F*_B_ bases in Fig. 1D, we use a bin width τ of 800 ps and the number of bins *N* of 1024. These parameters are chosen to optimize the JTIs in both the time and frequency bases. Each coincidence counting of the large Hilbert space is completed within 3 s, and no accidental subtraction is used.

We then conducted the validation of mutual unbiasedness of the two bases by using cross-basis measurements within our time-frequency bases. Two *d*-dimensional bases, denoted by *m* and *n*, are considered mutually unbiased when their constituent elements, denoted by *i* and *j*, adhere to the following relation (*39*, *46*)

$${|\langle t_{i}|d_{j}\rangle |}^{2}={|\langle \psi_{m,i}|\psi_{n,j}\rangle |}^{2}=\left\{\begin{array}{cc} \frac{1}{d} & \text{for}\;m\neq n\\ \delta_{\mathrm{ij}} & \text{for}\;m=n \end{array}\right.$$ (2)

for all *i* and *j*. *t_i_* is the temporal basis, and *d_j_* is the dispersive basis that is conjugate to the temporal basis. One should note here that the two bases span overlapping but not identical Hilbert spaces $\sum |t_{i}\rangle \langle t_{i}|\neq \sum |d_{i}\rangle \langle d_{i}|$. We should thus preface that all measurements are made under a double-fair sampling assumption: For one, we assume coincidences to be representative of the whole ensemble, even though singles are ignored and the correlations in temporal and frequency bases are, on average, representative of the correlations therein despite only sampling from smaller subspaces. The fact that the coincidences lead to MUB-consistent results still implies that the results obtained in one base give minimal information about the corresponding outcomes in the other basis. We verify their unbiased nature by measuring cross-detection probabilities. This involves using our cross-basis time-frequency and frequency-time measurements. Our verification results are given in Materials and Methods. The normalized Frobenius norm of the difference between the normalized time-frequency bases’ correlation matrix and the ideal correlation matrix for MUBs in *d* = 1021 is 0.05%. We summarized our cross-basis verification results for various dimensions, in which we certify entanglement and evaluate secure key rates, in the Materials and Methods.

**Fig. 2.**
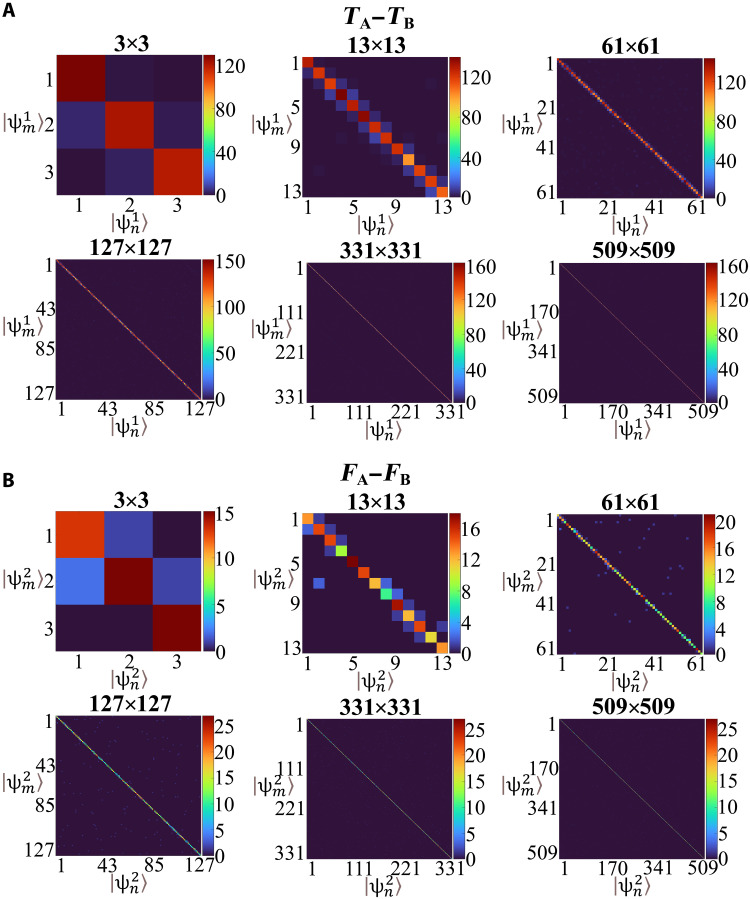
Experimental time-frequency bases up to 509 × 509 dimensions. (**A** and **B**) Experimental measured 3 × 3, 13 × 13, 61 × 61, 127 × 127, 331 × 331, and 509 × 509 Hilbert space dimensional JTI for temporal and frequency-resolved measurement bases. We can observe that our JTIs from both bases are dimensionally independent with respect to the measurements, owing to the large-alphabet arrival-time encoding, and the sufficient detected coincidence counts in our experimental setup. For all the measurements presented here, the JTI of the $T_{A}-T_{B}$ basis has higher diagonal coincidence counts than that of $F_{A}-F_{B}$ basis, which is mainly due to the losses in of the time-to-frequency converter (which is in total of ≈6 dB). From the same reason, we observe that the $F_{A}-F_{B}$ basis is noisier than the $T_{A}-T_{B}$ basis. For all experimental data in (A) and (B), the duration of measured coincidence counting is 3 s, and the raw data are presented here.

Figure 2 shows the coincidence measurement outcomes in the experimental time-frequency bases up to 509 × 509 dimensions, measured with a bin width τ of 800 ps and the number of bins *N* of 1024 in the two-shot measurements, akin to Fig. 1 (C and D). We illustrate here for experimental Hilbert spaces measured at prime numbers 3 × 3, 13 × 13, 61 × 61, 127 × 127, 331 × 331, and 509 × 509. We observe that our JTIs from both bases are scalable in measurement dimensions, with the dimensional measurement independence due to our large-alphabet arrival-time encoding approach, given that we have sufficient detected coincidence counts in our experimental setup, even up to 509 × 509 spaces. For all the measurements presented here, the JTI of the $T_{A}-T_{B}$ basis has higher diagonal coincidence counts than that of the $F_{A}-F_{B}$ basis because of the ≈3‐dB losses in each time-to-frequency dispersion module, and thus, the $F_{A}-F_{B}$ basis is noisier.

### Certification of high-dimensional entanglement

In this section, we certify and quantify the high-dimensional entanglement described by our experimental data that come from the scalable and scan-free JTI measurements in our scheme. Our approach is to use a fidelity-based Schmidt-number witness from (*39*) and a distillable entanglement bound from (*62*, *63*) [see also equation (17.135) in (*64*)] that requires measurements in (at least) two complementary bases. We apply these methods to the data obtained from our temporal and dispersive basis measurements. Let us first give a brief overview of both approaches and defer the more technical summary to Materials and Methods.

The first method (*39*) certifies the Schmidt number of a state ρ by estimating a fidelity lower bound $\tilde{F}\left(\rho ,\Phi \right)$ with respect to a pure target entangled state ∣Φ〉 (which we choose to be the maximally entangled state) with the maximum Schmidt rank *d*. If $\tilde{F}\left(\rho ,\Phi \right)$ exceeds the upper bound $B_{k}$, which we define in Materials and Methods, for all states with Schmidt number *k*, then ρ is certified to have a Schmidt number at least *k* + 1. In the following, the Schmidt number or the entanglement dimension *d*_ent_ of a state ρ will only be referred to the maximum Schmidt number that we can certify from ρ. Intuitively, a higher entanglement dimension *d*_ent_ enables more information to be encoded and transmitted securely (as we will see in the next section), making it a natural quantifier of high-dimensional entanglement.

The second method (*62*, *63*) lower bounds the distillable entanglement or entanglement of distillation (*E*_D_), which represents the maximum asymptotic average number of maximally entangled two-qubit states that can be extracted per copy of a quantum state ρ using classical communication and local operations (*26*, *65*). For a pair of two-dimensional quantum systems, the maximum entanglement they can have is 1 ebit, which corresponds to a Bell state. In contrast, higher-dimensional systems can potentially contain up to log(*d*) bits of entanglement, thereby enabling certification in high-dimensional scenarios. The bound uses the respective conditional Shannon entropies of the measurement outcomes in the first and second bases, as well as the maximum overlap between the two bases (which would be 1/*d* in the case of ideal MUBs, as presented in the prior section). With limited counts, one expects individual elements to deviate statistically from the mean, thus rendering the determination of the maximum overlap of the two bases a challenge. We work with the hypothesis of mutual unbiasedness, which we test and see that the expected deviation is in line with purely statistical fluctuations around the mean (more details are provided in Materials and Methods).

**Fig. 3.**
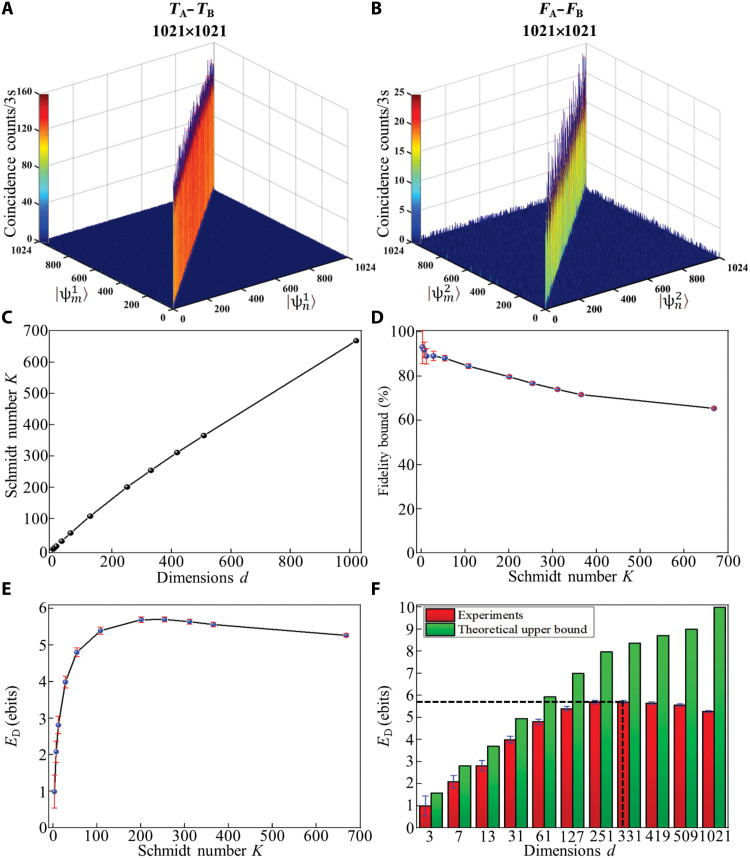
High-dimensional entanglement certification from a maximum of 1021-dimensional JTI measured in the temporal and frequency bases. (**A** and **B**) Coincidence counts for measuring in the $T_{A}-T_{B}$ and $F_{A}-F_{B}$ bases in a two-shot setting, with consistent bin width τ and number of bins *N*, as shown in Figs. 1 and 2. The timing for coincidence counting is 3 s, and the data are reported without accidental subtractions. The strong correlations in both measurement bases signify robustness of large-alphabet arrival-time encoding. (**C** and **D**) The certified Schmidt number *k* for each measured local dimension *d* and their associated lower bound of the fidelity $\tilde{F}\left(\rho ,\Phi \right)$ with respect to the maximally entangled state are shown. The maximum certified Schmidt number is 668 at a fidelity of 65.4 ± 0.4% in *d* = 1021. (**E** and **F**) The distillable entanglement *E*_D_ for various local dimensions *d* is shown together with the certified Schmidt numbers and the theoretical upper bound of *E*_D_, log_2_(*d*).

Before we present the entanglement certification results, let us first demonstrate the scalability of our JTI measurements performed in the time-frequency bases. In Fig. 3 (A and B), we present the biphoton coincidence counts from 1021 × 1021–dimensional discretized JTI measurements of the time- and frequency-resolved measurement bases, with consistent bin width τ and number of bins *N*, as described in Figs. 1 and 2. Even up to a local Hilbert space dimension of 1021, strong quantum temporal correlations are observed in both measurement bases. This represents the robustness of large-alphabet arrival-time encoding, and such measurements are obtained in a two-shot setting. Note that in Fig. 3 (A and B), the duration of measured coincidence counting is 3 s, and the raw experimental data are presented without any accidental subtractions.

We now move on to discuss our entanglement certification results for different input dimensions, which are shown in Fig. 3 (C to F). From the full 1021 × 1021–dimensional Hilbert space corresponding to the discretized JTI measurements, we can certify an entanglement dimension *d*_ent_ up to 668 and a distillable entanglement of $E_{D}=5.27\pm 0.04$ ebits with the two-shot time-frequency basis measurements. The maximum distillable entanglement *E*_D_ of 5.70 ± 0.07 ebits is achieved when the entangled dimension *d*_ent_ is 246 (where the local dimension *d* is 331). We attribute the discrepancy in the local dimensions at which the two quantities achieve maximum to their different noise sensitivity, as higher-dimensional measurements tend to be noisier. We also compare our experimental certified *E*_D_ with the theoretical upper bound of log(*d*) in Fig. 3F and observe the same falling behavior in *E*_D_, which suggests noise in the two-shot JTI measurements as *d* grows. We further support this observation with Fig. 3D, where we show that the lower bound on the state fidelity $\tilde{F}\left(\rho ,\Phi \right)$ reaches the minimum at 65.4 ± 0.4% for *d* = 1021. The uncertainty in the fidelity is calculated on the basis of the assumption that the measured frequency of each measurement outcome is the mean value of a Poisson distribution. By sampling these distributions of all outcomes jointly for 1000 to 2000 times and assuming a final Gaussian distribution for the computed fidelities, the error bars are taken to be 3 standard deviations from the observed fidelity. We remark that both the certified entanglement dimension *d*_ent_ and distillable entanglement *E*_D_ are record measurements to date. For a comprehensive comparison with known results, please refer to Table 1.

**Table 1.** Comparison of assumptions used in various high-dimensional quantum photonic experiments. N/A: information not available or not applicable.

Experiments	(*37*)	(*46*)	(*82*)	(*26*)	(*39*)	(*47*)	(*44*)	(*48*)	(*29*)	(*20*)	(*83*)	This work
Domains	OAM	Pixel	Energy-time-polarization	Energy-time	OAM	Pixel	Path	Pixel	Energy-time	Frequency	Time-bin	Energy-time
Years	2014	2017	2017	2017	2018	2019	2020	2020	2021	2022	2025	2025
Certified entangled dimensions	100*	3	4	18	9	10	32	97	4	8	16	668
*E*_D_ (ebits)	N/A	3.05	1.47	4.1†	N/A	3.43‡	3.73	4.0	1.89†	2.32	1.992	5.701

* Conservation of orbital angular momentum (OAM).

† No cross-talk in the computational basis.

‡ Raw data without accidental subtraction.

The results in Figs. 1 to 3 clearly demonstrate the advantage of our approach, which is attributable to the following techniques. Using SNSPDs with reduced timing jitter in the telecom wavelengths (*60*, *61*) allows us to use smaller bin widths τ such that we are able to encompass the entire temporal correlation peak. Simultaneously, we can steadily increase the number of bins *N* to expand the dimensionality of our discretized JTIs. We note that the optimal parameters for JTIs presented in this work can be adapted to other quantum photonic systems by considering the corresponding FWHM of temporal correlation peaks, coincidence counts of the SPDC source, loss of telecom components, efficiency, and timing jitter of the detectors. This multibit temporal encoding scheme ensures that the number and duration of measurements remain constant over the subspace dimension for our discretized JTIs. We illustrate this feature across various temporal subspaces in Figs. 2 and 3. Therefore, our approach also offers significantly faster measurements, with an acquisition time three orders of magnitude faster for 61-dimensional data and six orders of magnitude faster for 1021-dimensional data. We summarize the comparison of the required number of local projective measurements versus different dimensions *d* for various techniques in Materials and Methods. In addition, having smaller bin widths τ and a larger number of bins *N* is preferable for achieving a higher key capacity in temporal encoding with large alphabets (*57*–*59*).

### Large-alphabet QKD

After successfully generating and certifying high-dimensional entanglement within our time-frequency (approximate) MUBs, we demonstrate one of the key applications of quantum photonic qudits: large-alphabet (*32*, *55*–*58*) QKD (*66*, *67*). Transmitting delicate quantum correlations through a noisy channel poses a substantial hurdle in quantum communication tasks (*5*, *6*, *68*). High-dimensional QKD protocols address this by encoding dense information in entangled biphoton states, enabling high key throughput (*32*, *57*) with enhanced robustness against detector dead time and environmental noise (*32*, *56*–*58*, *69*). Different trust models exist for QKD (*5*, *68*, *70*), ranging from fully device-trusted prepare-and-measure schemes to device-independent protocols that rely on loophole-free Bell violations. Entanglement-based QKD represents a reasonable balance between these extremes: It provides security against coherent attacks, is readily certifiable, avoids the need for specialized countermeasures such as decoy-state methods, and still achieves competitive key rates in realistic implementations.

By combining recent advances in high-dimensional protocols and coherent composable finite-size security proofs, originally developed for Franson-certified time-bin experiments, we generalize the protocol and provide a comprehensive security analysis of high-dimensional, finite-size protocols that are based on two MUBs. Crucially, the actual phase relation between the two bases does not need to be known or assumed, only the relative overlap. This is inherited from the witness used in the security proof, which is invariant under relative phase transformations. For the overlaps, we performed cross-basis measurement, certifying a good correspondence of the ideal positive operator-valued measure with the experimental implementation, subject to a fair sampling assumption. In more detail, we use the measured coincidence-click matrices to determine the observed average of the observable $\hat{W}=\sum_{i=0}^{d-1}A_{F}^{i}⊗B_{F}^{i}$ with ${\left\{A_{F}^{i}\right\}}_{i}$ and ${\left\{B_{F}^{i}\right\}}_{i}$ being Alice and Bob’s frequency basis measurements, respectively. In line with the assumptions of device-dependent QKD, we assume that the measurement devices are in Alice and Bob’s trusted laboratories, hence under their control. In particular, this means that we assumed that the theoretical positive operator-valued measure elements are also practically implemented (up to relative phases). While a good alignment between the theoretical description and the practical implementation could be experimentally verified, quantifying deviations between the theoretical model and the practical implementation is still an active area of research, even for qubit-based protocols (*71*, *72*). Thus, although beyond the scope of the present proof-of-principle work, bounding the influence of small deviations from theoretical measurements remains an interesting avenue for future research. On the basis of our witness-based approach, as we detail in Materials and Methods, we certify a record asymptotic key rate using only short measurement times and statistics. In addition, we use a variable-length security argument (*73*) to demonstrate the potential for a composable finite-size secure key rate in realistic measurement times.

**Fig. 4.**
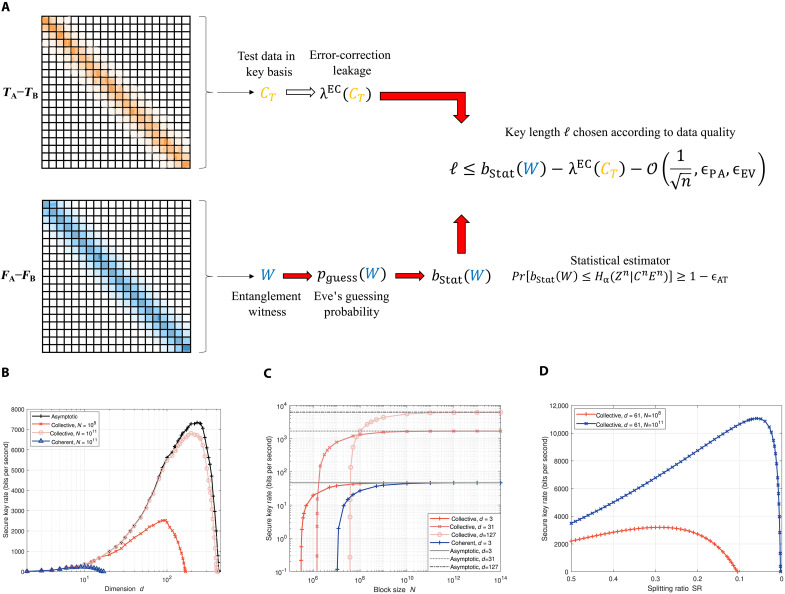
Composable security large-alphabet quantum communication via time-frequency bases. (**A**) Illustration of the security argument: On the basis of the recorded coincidence-click matrices, we derive two quantities. First, from the FF-clicks, we derive the expectation of an entanglement witness, which allows us to bound Eve’s guessing probability on the final key, which, in turn, allows us to derive a high-probability lower bound on Alice and Bob’s private entropy. Second, from the disclosed TT-clicks, we derive the error-correction leakage. The difference of those yields (up to second-order correction terms) a reliable high-probability lower bound on the secure key length. (**B**) Secure key rate in bits per second versus system dimension in four different scenarios: asymptotic (black pluses), i.i.d. collective attacks with block size *N* = 10^8^ (orange crosses), i.i.d. collective attacks with block size *N* = 10^11^ (peach circles), and coherent attacks with *M* = 10^11^ (blue triangles). On the basis of our data, we obtain optimal system dimensions of $d_{\text{opt}}^{\text{asym}}\left(\infty \right)=232$ for the asymptotic scenario, $d_{\text{opt}}^{\text{coll}}\left(10^{8}\right)=96$ for i.i.d. collective attacks with *N* = 10^8^, $d_{\text{opt}}^{\text{coll}}\left(10^{11}\right)=196$ for i.i.d. collective attacks with *N* = 10^11^, and $d_{\text{opt}}^{\text{coh}}\left(10^{11}\right)=9$ for coherent attacks with *N* = 10^11^. (**C**) Secure key rate in bits per second versus block size *N* for three different dimensions. In all three cases, the curves converge to the asymptotic rates (horizontal lines) already for practically viable block sizes. (**D**) Examination of the secure key rate in bits per second versus splitting ratio between time and frequency measurements for two different block sizes and fixed dimension *d* = 61.

While fixed-length security arguments, which are predominant in the literature and build security around the expected behavior of the quantum channel connecting Alice and Bob, we follow an adaptive-length approach and build the security argument around the observed statistics. For fixed-length approaches, the expected channel behavior needs to be fixed before the protocol execution. After the protocol run, one performs an acceptance test, where the observed statistics are compared to the predefined expected behavior. In case the test accepts, the protocol produces a key of a fixed length; otherwise, the protocol aborts and does not produce key at all. This is quite restrictive and, in practice, leads to an excessive number of aborted rounds. We circumvent this problem and build our security argument around the observed statistics. In more detail, we adapt the variable-length approach (*74*) for HD-QKD protocols from (*73*). However, we replace the witness-inspired completion technique (*75*) by data obtained from mutually unbiased basis measurements. Thus, we directly observe the correlation between Alice and Bob’s test rounds in the frequency basis $W=\sum_{i=0}^{d-1}P\left(\mathrm{ii}|\text{FF}\right)$, where P(ii∣FF) is the probability that Alice and Bob obtain equal outcomes when both measure in the frequency basis (*76*). On the basis of this observation, we find a statistical estimator $b_{\text{stat}}\left(W\right)$, which is a high-probability lower bound on the private entropy given Eve’s side information. The details of the security argument are illustrated in Fig. 4A. For further details, we refer to Materials and Methods.

We use this approach to illustrate the key rate potential of our setup. We note that in line with existing works on the implementation of QKD protocols, our analysis conditions on coincidence clicks. Under the fair sampling assumption, those conditioned rounds are representative of the full *N*-round quantum state. While this remains an assumption in the present work, which is a proof of principle, it does not represent a fundamental obstacle and can be removed in future works. Besides conditioning on coincidence clicks, we do not perform any additional postselection (e.g., accidental subtraction), which, in principle, could further enhance the key rate but would require careful treatment in the security analysis. In Fig. 4B, we plot the secure key rate per second over the dimension of the underlying quantum system. For the asymptotic data, we observe a peak for *d* = 232, while for *N* = 10^11^, the inferred collective attack key rate peaks only slightly below at *d* = 196. Even for *N* = 10^8^, we observe a peak for *d* = 96 and, therefore, a clear outperformance of high-dimensional QKD versus qubits. This holds even true for coherent attacks. As we illustrate in Fig. 4C, our finite-size rates approach the asymptotic limit already for relatively small and, therefore, realistic block sizes. All results so far referred to our standard setting, where Alice and Bob measure in both bases with equal probability. However, as we argue in Fig. 4D, this is far from optimal. Optimizing this splitting ratio can boost the key rates further, as showcased for block size *N* = 10^11^, where the key rates can be increased by a factor of 3 compared to 50:50 splitting. Optimizing over both dimension and splitting ratio, we certify a composable collective independently and identically distributed (i.i.d.) key rate potential of 15.6 kilobits (kB)/s for *d* = 232 with a splitting ratio of 14%. The primary goal of this section was to demonstrate the key rate potential of the platform and measurement method in use and to provide a comparison with qubit protocols. This included optimizations over the chosen dimension and the splitting ratio, as well as the examination of the key rate for two different levels of security and across different block sizes. As shown in Fig. 4D, we find an optimal splitting ratio of 29% for *N* = 10^11^, while for *N* = 10^11^, the optimal splitting ratio is found to be 6%. This highlights the fact that optimal splitting is far below the default 50% and the key rates can be improved significantly by optimizing this parameter. While such considerations are essential for understanding the behavior and the key rate potential of the setup, for continuous operation, one would usually fix those parameters before protocol execution on the basis of the hardware characteristics (e.g., source brightness and postprocessing capabilities) and an estimate of the channel behavior (loss and noise). However, given that the expected characteristics of the whole system do not enter the adaptive security argument, deviations thereof do not compromise security.

## DISCUSSION

In this study, we presented an approach for reconstructing the temporal structure of correlated biphoton quantum states. Our proposal leverages the large-alphabet time-bin encoding of SPDC photon pairs and uses low-jitter single-photon detectors to probe the arrival time of the qudit states. Only two measurements are required to reconstruct the JTI of biphoton entangled states with high fidelity. We concentrated on the case of SPDC, generated from a nonlinear waveguide, and analyzed the temporal and frequency-resolved correlations, high-dimensional energy-time entangled biphoton states, and large-alphabet QKD in a telecom fiber link. The results demonstrate the superiority of this technique over projective techniques [such as in (*39*, *46*–*48*)] for benchmarking highly correlated quantum states. We observe that performing a projective measurement on a 1021-dimensional subspace would require several days to accumulate the necessary statistics for 1021^2^ (or ≈2^19.99^) projections. This extended duration is due to the low count rates associated with the lossy techniques used for mode projection. In contrast, our approach enables us to gather the required data within a few seconds, regardless of the subspace dimensionality being analyzed (with the only limitation being the detectable coincidence counts in our experimental system). We certified 668-dimensional entanglement at *d* = 1021 and distillable entanglement of up to 5.70 ± 0.07 ebits at *d* = 331 through maintaining high fidelities with the maximally entangled state states with a minimum of 65.4 ± 0.4% for *d* = 1021.

Here, in addition to high-dimensional entanglement certifications, we extended recent QKD protocols to demonstrate an exemplary quantum communication experiment. We developed a composable finite-size security proof tailored toward the two measurements and based upon (*76*), proving the capacity for a secret key of 15.6 kB/s. Our adaptable approach uses optical fiber components commonly used in telecom wavelengths, alongside the recent low-jitter (*61*) single-photon detectors. These numbers can be further improved by the continuous advancement of SPDC sources, telecom fiber components, and low-jitter (*60*, *61*) and highly efficient SNSPDs.

Besides the high-dimensional time-bin encoding, another key ingredient is to generate frequency-resolved temporal correlations with time-to-frequency converters. Future studies will focus on extending this platform to various biphoton and multiphoton states, produced from separate distance sources. The results could pave the way for scalable high-dimensional quantum information processing as well as robust high-rate quantum communication networks toward the fully deployable quantum internet.

## MATERIALS AND METHODS

### Experimental details

We use a continuous-wave distributed Bragg reflector single-frequency laser (Thorlab DBR780PN) to pump a type II phase-matched, single-spatial-mode periodically poled potassium titanyl phosphate waveguide (AdVR Inc.) at 1560 nm. A fiber polarization controller (FPC) positioned before the periodically poled potassium titanyl phosphate waveguide optimizes the generation of orthogonally polarized SPDC photons. Residual pump photons are removed using a longpass filter (LPF) and an angle-mounted bandpass filter (BPF) with 95% passband transmission (Semrock NIR01-1570/3). Last, a polarizing beam splitter (PBS) separates the signal and idler photons, directing them to Alice and Bob. Then, we implement the random choice of measurements between temporal basis ($T_{A}-T_{B}$) and frequency-resolved basis measurements ($F_{A}-F_{B}$) with 50:50 fiber beam splitters. This symmetric configuration guarantees ample coincidence counts to establish time-frequency MUB measurements. The $T_{A}-T_{B}$ bases correspond to direct detection of photon arrival time from both parties, and the $F_{A}-F_{B}$ bases are the dispersive basis that is mutually unbiased with respect to the temporal states. For both measurements, we use arrival-time high-dimensional encoding. Alice and Bob independently measure the photon arrival times. Both parties use *N* consecutive time bins to form a time frame. For frequency-resolved measurements, we use a pair of large dispersion modules, with ±10,000 ps/nm DEM (DCM), and each of them has a loss of only 3 dB (Proximion). The effective frequency resolution in our experiments can be obtained as FWHM timing jitter divided by the applied dispersion, which is 0.00329 nm (0.41 GHz), sizably smaller than the FWHM bandwidth of our SPDC source (250 GHz).

The coincidence counts from the $T_{A}-T_{B}$ bases are recorded by two low-jitter SNSPDs (*61*). Recently, impedance-matched differential SNSPDs have been developed to simultaneously achieve a practical active area for efficient coupling to a single-mode fiber and low-jitter operation. The two detectors used in this work featured optical stacks with a double antireflection coating above the nanowire, optimized for 1550 nm, resulting in ~80% efficiency at this wavelength and a timing jitter of around 13.1 ps. Impedance matching in SNSPDs significantly improves the signal-to-noise ratio of the readout, with system timing jitter around 15 ps. Using these low-jitter SNSPDs and our coincidence counting module (Picoharp 300), we observed a temporal cross-correlation peak with an FWHM of ~32.9 ps, as shown in the inset of Fig. 1C. The broadening of the FWHM of the cross-correlation peak is due to the electronic jitter of our coincidence counting module. In the future, it is conceivable that we could enhance detector jitter by using quicker superconducting materials and advancements in nanofabrication, potentially enabling the resolution of temporal correlations of SPDC photons at the fundamental limit of two-photon correlation time. On the other hand, for frequency-resolved measurements, we register coincidence counts from *F*_A_ and *F*_B_ bases via low-jitter SNSPDs. Here, we observed a temporal cross-correlation peak with an FWHM of ~125.5 ps, as shown in the inset of Fig. 1D. In this case, the broadening of the FWHM of the cross-correlation peak is due to the electronic jitter of our coincidence counting module and the imperfect nonlocal dispersion cancellation of our large dispersion modules. In Table 2, we provide comprehensive characterization of the optical loss for the whole measurement setup in the main text (Fig. 1B). The dominant factors limiting the performance of high-dimensional entanglement certification and high-dimensional QKD are the detection jitter, dispersion imperfections, and optical loss of the system.

**Table 2.** Characterized sources of optical loss in the experimental setup.

Source of loss	Loss in dB
SPDC output fiber coupling	3
Fiber bench	0.97
Longpass filter (LPF)	0.8
Bandpass filter (BPF)	0.2
Fiber polarization controllers (FPCs)	3 (1 each)
PBS	1.1
Fiber connector loss	1
Fiber BS	3 each
DCM	3.67
DEM	2.61
Low-jitter detectors	2 (1 each)

### High-dimensional entanglement witness

In this section, we provide more details of the certification techniques we use to observe high-dimensional entanglement (*39*). To begin, we examine the certification process for high-dimensional entanglement in a bipartite quantum system, where the total Hilbert space $H_{\text{AB}}=H_{A}⊗H_{B}$ of an a priori unknown quantum state ρ has local dimensions $\text{dim}H_{A}=\text{dim}H_{B}=d$. To certify the Schmidt number (or the entanglement dimension) of ρ, we consider the fidelity F(ρ,Φ) with respect to the target quantum state ∣Φ〉, which takes the form

$$F\left(\rho ,\Phi \right)=\text{Tr}\left(|\Phi \rangle \langle \Phi |\rho \right)=\sum_{m,n=0}^{d-1}\lambda_{m}\lambda_{n}\langle \mathrm{mm}|\rho |\mathrm{nn}\rangle$$ (3)

with λ*_n_* being the corresponding Schmidt coefficients for the target quantum state ∣Φ〉. The entanglement dimension can be lower bounded by considering the following inequality, which holds for any quantum state ρ with Schmidt number at most k≤d

$$F\left(\rho ,\Phi \right)\leq B_{k}\left(\Phi \right)≔\sum_{m=0}^{k-1}\lambda_{m}^{2}$$ (4)

with m∈{0,…,d−1} such that $\lambda_{m}\geq \lambda_{m^{\prime }}\;\forall \;m<m^{\prime }$. Hence, any quantum state with $F\left(\rho ,\Phi \right)>B_{k}\left(\Phi \right)$ is incompatible with a Schmidt number of *k* or less, thereby certifying a minimum entanglement dimension of *k* + 1.

Therefore, the subsequent step involves experimentally determining the fidelity of the quantum state F(ρ,Φ). We use the respective matrix elements to lower bound the fidelity of the target state F(ρ,Φ) by first separating it into two parts, $F\left(\rho ,\Phi \right)=F_{1}\left(\rho ,\Phi \right)+F_{2}\left(\rho ,\Phi \right)$, with

$$F_{1}\left(\rho ,\Phi \right)≔\frac{1}{d}\sum_{m}\langle \mathrm{mm}|\rho |\mathrm{mm}\rangle$$ (5)

$$F_{2}\left(\rho ,\Phi \right)≔\frac{1}{d}\sum_{m\neq n}\langle \mathrm{mm}|\rho |\mathrm{nn}\rangle$$ (6)

if the target state is the maximally entangled state $|\Phi \rangle =\frac{1}{\sqrt{d}}\sum_{m=0}^{d-1}|\mathrm{mm}\rangle$. The part $F_{1}\left(\rho ,\Phi \right)$ can be directly extracted from measurements in one basis, while the part $F_{2}\left(\rho ,\Phi \right)$ can be lower bounded by ${\tilde{F}}_{2}\left(\rho ,\Phi \right)$ using measurements in an additional basis, where

$$\begin{array}{c} {\tilde{F}}_{2}≔\sum_{j}\langle {\tilde{j}\tilde{j}}^{*}|\rho |{\tilde{j}\tilde{j}}^{*}\rangle -\frac{1}{d}\sum_{m,n}\langle \mathrm{mn}|\rho |\mathrm{mn}\rangle \\ -\sum_{m\neq m^{\prime },m\neq ,n\neq n^{\prime },n^{\prime }\neq m^{\prime }}{\tilde{\gamma }}_{mm^{\prime }nn^{\prime }}\sqrt{\langle m^{\prime }n^{\prime }|\rho |m^{\prime }n^{\prime }\rangle \langle \mathrm{mn}|\rho |\mathrm{mn}\rangle } \end{array}$$ (7)

where ${\tilde{\gamma }}_{mm^{\prime }nn^{\prime }}$ is given by

$${\tilde{\gamma }}_{mm^{\prime }n^{\prime }}=\left\{\begin{array}{cc} 0, & \text{if}\;\left(m-m^{\prime }-n+n^{\prime }\right){\text{mod}}_{d}\neq 0,\\ \frac{1}{d}, & \text{otherwise} \end{array}\right.$$ (8)

Therefore, by measuring in two different bases, we can constrain the fidelity term $F_{2}\left(\rho ,\Phi \right)$; this, in turn, provides a lower bound $\tilde{F}\left(\rho ,\Phi \right)$ for the fidelity F(ρ,Φ) in the Schmidt-number witness inequality presented in Eq. 4, resulting in the following relationship that relates the fidelity lower bound to the upper bound for states with Schmidt number *k*

$$\tilde{F}\left(\rho ,\Phi \right)\leq F\left(\rho ,\Phi \right)\leq B_{k}\left(\Phi \right)$$ (9)

By using this inequality as a witness, the entanglement dimension *d*_ent_ that is certifiable is the maximal *k* such that $\tilde{F}\left(\rho ,\Phi \right)\geq B_{k}\left(\Phi \right)$.

### Entanglement of distillation

Next, we describe how we can lower bound the *E*_D_ in our quantum systems using two measurement bases. First, let us recall the definition of the conditional Shannon entropy

$$H\left(A_{i}|B_{i}\right)=H\left(\left\{p_{\mathrm{jk}}^{\left(i\right)}\right\}\right)-H\left(\left\{p_{j}^{\left(i\right)}\right\}\right)$$ (10)

where $p_{\mathrm{jk}}^{\left(i\right)}=\langle j^{\left(i\right)}k^{\left(i\right)}|\rho {|j^{\left(i\right)}k^{\left(i\right)}\rangle }_{\text{AB}}$ and $p_{j}^{\left(i\right)}=\sum_{k}\langle j^{\left(i\right)}k^{\left(i\right)}|\rho {|j^{\left(i\right)}k^{\left(i\right)}\rangle }_{\text{AB}}$, with *i* being the basis label. Knowing that these terms are tied to coincidence counts measured in any two bases, we can bound the distillable entanglement *E*_D_ from below with (*62*–*64*)

$$E_{D}\geq -{\text{log}}_{2}\left(\underset{i,j}{\text{max}}{|\langle i|\tilde{j}\rangle |}^{2}\right)-H\left(A_{1}|B_{1}\right)-H\left(A_{2}|B_{2}\right)$$ (11)

where ${\text{max}}_{i,j}{|\langle i|j\rangle |}^{2}$ is the maximal overlap of elements of the two bases used (which would be 1/*d* in case of ideal MUBs, as presented in the main text).

### Comparison of the required number of local measurement settings versus different local dimensions *d* for different techniques

**Fig. 5.**
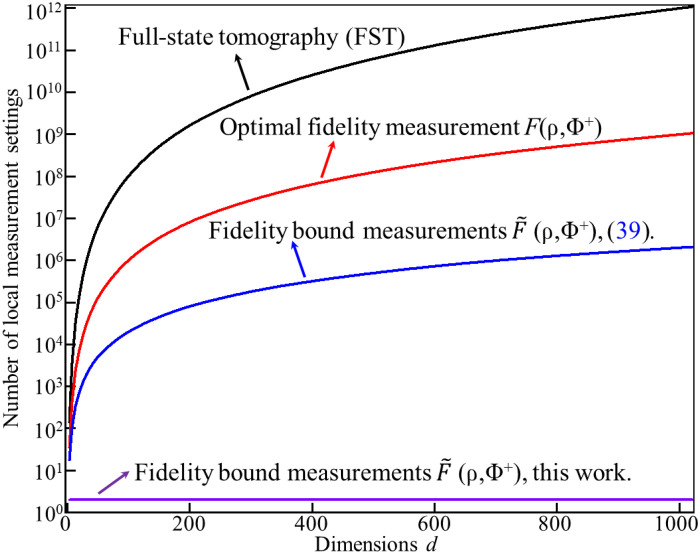
Comparison of the required number of local projective measurement settings in different local dimensions *d* for different techniques. In this work, we highlight the constant number of measurement settings, given that we only need a single setting for each of the $T_{A}-T_{B}$ and $F_{A}-F_{B}$ bases, independent of the dimensions *d*. Hence, our work represents many orders-of-magnitude improvement over traditional FST and prior literature (*39*, *46*, *48*, *77*). We note that there are only a few fundamental limitations of our scheme: the number of measurable coincidence counts from the photon-pair source, loss in the time-to-frequency converter and other fiber components, and the timing jitter and detection efficiency of accessible single-photon detectors.

Extended data (Fig. 5) compare the required number of local projective measurement settings used in this work with those required by other techniques across different dimensions *d*. For FST, ${\left(d+1\right)}^{2}d^{2}$ local projective measurement settings are required (*39*, *77*), while optimal fidelity measurement F(ρ,Φ) requires $\left(d+1\right)d^{2}$ such measurements (*39*). More recently, it has been reported that only two measurement bases and 2*d*^2^ local projective measurement settings are sufficient to certify high-dimensional entanglement with fidelity bounds F(ρ,Φ) (*39*). In this work, we highlight that only a constant number of measurement settings are required, given that a single setting is sufficient for each of the $T_{A}-T_{B}$ and $F_{A}-F_{B}$ bases, independent of the dimensions *d*. Hence, our work represents many orders-of-magnitude improvement over traditional FST and prior literature (*39*, *46*, *48*, *77*). For example, at *d* = 1021, FST needs ≈10^12^ local projective measurement settings, and prior work using the fidelity bound method needs ≈10^6^ settings (*39*), whereas our method requires only two measurement settings to certify high-dimensional entanglement of the quantum photonic state. We note that there are only a few fundamental limitations of our scheme: the number of measurable coincidence counts from the photon-pair source, loss in the time-to-frequency converter and other fiber components, and the timing jitter and detection efficiency of accessible single-photon detectors.

### Composable security analysis

We adapt the adaptive-length (*74*) security argument from (*73*). However, instead of using the witness-completion approach from (*75*), we directly exploit the MUB measurement results and observe the correlation between Alice and Bob’s test rounds in the frequency basis $W=\sum_{i=0}^{d-1}P\left(\mathrm{ii}|\mathrm{FF}\right)$, where P(ii∣FF) is the probability that Alice and Bob obtain equal outcomes when both measure in the frequency basis (*76*). On the basis of this observation, we find a statistical estimator $b_{\text{stat}}\left(W\right)$, which is a high-probability lower bound to the Rényi entropy $H_{\alpha }{\left(Z^{n}|C^{n}E^{n}\right)}_{\rho }$ of the underlying quantum state

$$\text{Pr}\left[b_{\text{stat}}\left(W\right)\leq H_{\alpha }{\left(Z^{n}|C^{n}E^{n}\right)}_{\rho }\right]\geq 1-\epsilon_{\text{AT}}$$ (12)

Here, *Z* is the key register, *C* is the transcript of the classical communication, *E* denotes Eve’s side information, and *n* is the number of key rounds. To find such an estimator, we need to construct a set *V*(*W*), which contains the unknown quantum state ρ with high probability, $\text{Pr}\left[\tau_{\text{AB}}\in V\left(W\right)\right]\geq 1-\epsilon_{\text{AT}}$. Let *o_W_* be the observed statistics for observable $\hat{W}=\sum_{i=0}^{d-1}A_{F}^{i}⊗B_{F}^{i}$ with ${\left\{A_{F}^{i}\right\}}_{i}$ and $\left\{B_{F}^{i}\right\}$ being Alice and Bob’s frequency basis measurements, we obtain

$$V\left(W\right)=\left\{\sigma \in D\left(H_{A}⊗H_{B}H_{E}:\right.|\text{Tr}\left[\hat{W}\sigma \right]-o_{W}|\leq \mu \right\}$$ (13)

where we obtain μ from Hoeffding’s inequality (*78*, *79*)

$$\mu =\sqrt{\frac{2{\left\|\hat{W}\right\|}_{\infty }^{2}}{k_{W}}\text{ln}\left(\frac{2}{\epsilon_{\text{AT}}}\right)}$$ (14)

Here, ϵ_AT_ is the security parameter associated with the statistical estimation procedure, and *k_W_* is the number of rounds used to test $\hat{W}$. Then, the statistical estimator reads

$$\begin{array}{cc} b_{\text{stat}}\left(W\right)≔ & n\underset{\tau_{\text{AB}}\in V\left(W\right)}{\text{min}}H_{\text{min}}{\left(X|E\right)}_{\Phi_{\text{var}}\left(\tau_{\text{ABE}}\right)}\\ & -n\left(\alpha -1\right){\text{log}}_{2}^{2}\left[\text{dim}\left(X\right)+1\right] \end{array}$$ (15)

where $1<\alpha <1+\frac{1}{{\text{log}}_{2}\left[2\text{dim}\left(X\right)+1\right]}$.

In addition, on the basis of the observation and the communication transcript, we determine the error-correction leakage $\lambda^{\text{EC}}\left(W,C\right)$. Then, the protocol conditioned on observing *W* during the statistical testing procedure and conditioned on passing error verification hashes to a key length of

$$\ell \left(W\right)≔\text{max}\left\{0,b_{\text{stat}}\left(W\right)-\lambda^{\text{EC}}\left(W\right)-\theta \left(\alpha ,\epsilon_{\text{PA}},\epsilon_{\text{EV}}\right)\right\}$$ (16)

where $\theta \left(\alpha ,\epsilon_{\text{PA}},\epsilon_{\text{EV}}\right)≔\frac{\alpha }{\alpha -1}\left[{\text{log}}_{2}\left(\frac{1}{4\epsilon_{\text{PA}}}+\frac{2}{\alpha }\right)\right]+\left\lceil {\text{log}}_{2}\left(\frac{1}{\epsilon_{\text{EV}}}\right)\right\rceil$; using $\lambda^{\text{EC}}\left(W,C\right)$ bits for error correction is ϵ_EV_-correct and is $\epsilon_{\text{AT}}+\epsilon_{\text{PA}}$-secure against i.i.d. collective attacks.

Using the postselection technique (*80*, *81*), we can lift security to general attacks. Once we proved security against collective attacks with the security parameter $\epsilon_{\text{PE}}+\epsilon_{\text{AT}}$ and the correctness parameter ϵ_EV_ conditioned on obtaining ${\vec{F}}^{\text{obs}}$ during acceptance testing and passing the error verification, the protocol is secure against coherent attacks with the security parameter $g_{n,x}\left[\sqrt{8\left(\epsilon_{\text{PE}}+\epsilon_{\text{AT}}\right)}+\frac{\tilde{\epsilon }}{2}\right]$, if the key is hashed to a length of

$$\begin{array}{c} \ell \left({\vec{F}}^{\text{obs}}\right)≔\\ \text{max}\left\{0,b_{\text{stat}}\left({\vec{F}}^{\text{obs}}\right)-\lambda^{\text{EC}}\left({\vec{F}}^{\text{obs}}\right)-\theta \left(\alpha ,\epsilon_{\text{PA}},\epsilon_{\text{EV}}\right)-2{\text{log}}_{2}\left(g_{n,x}\right)-2{\text{log}}_{2}\left(\frac{1}{\tilde{\epsilon }}\right)\right\} \end{array}$$ (17)

where $g_{n,x}=\left(\begin{array}{c} n+x-1\\ n \end{array}\right)$ for $x=d_{A}^{2}d_{B}^{2}$ and $\tilde{\epsilon }>0$ can be chosen freely.

Thus, it remains to determine the statistical estimator, i.e., to solve ${\text{min}}_{\tau_{\text{AB}}\in V\left(W\right)}H_{\text{min}}{\left(X|E\right)}_{\Phi_{\text{var}}\left(\tau_{\text{ABE}}\right)}$. The present setup performs two MUB measurements; hence, we may replace the semianalytic dual method from (*75*), designed for evaluating *H*_min_ arbitrary setups, by a generalized version of the technique introduced in (*76*), which exploits the high symmetry of mutually unbiased basis measurements. Consequently, we obtain for the statistical estimator

$$H_{\text{min}}{\left(Z|E\right)}_{\rho }={\text{log}}_{2}d-2{\text{log}}_{2}\left[\sqrt{W-\mu }+\sqrt{\left(d-1\right)\left(1-W+\mu \right)}\right]$$ (18)

We applied our security argument to the observed data. Therefore, we chose $\epsilon_{\text{EC}}=\epsilon_{\text{PA}}=\epsilon_{\text{AT}}=\frac{1}{2}\times 10^{-10}$ and $\alpha =1+\frac{1}{\sqrt{n}}$, leading to a total security parameter of $\epsilon_{\text{sec}}=10^{-10}$.

### Cross-basis time-frequency measurements

**Fig. 6.**
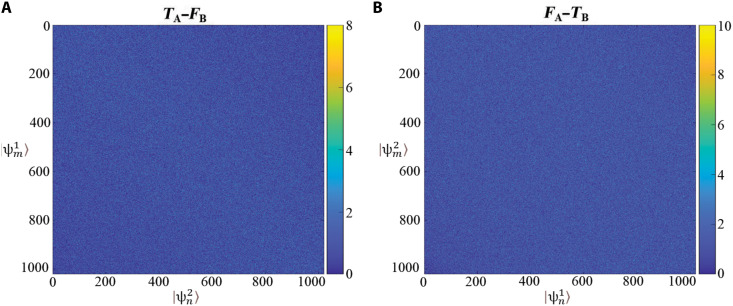
Time-frequency cross-basis measurements with a dimension of 1021. (**A** and **B**) A 31-dimensional discretized JTI from *T*_A_ and *F*_B_ and *F*_A_ and *T*_B_ bases, respectively. The bin width τ and number of bins *N* are 10 ps and 1021, respectively. We can observe the near-uniform JTI with low coincidence counts. Here, the duration of measured coincidence counting is 3 s, and no subtraction of background or accidental counts is performed.

Here, we provide the experimental results and theoretical analysis of *T*_A_ and *F*_B_ and *F*_A_ and *T*_B_ bases. The A and B refer to Alice and Bob, respectively. For the cross-basis measurements, we perform them with the experimental setup in the main text (Fig. 1B). Figure 6 (A and B) shows the measured 1021-dimensional discretized JTI from *T*_A_ and *F*_B_ and *F*_A_ and *T*_B_ bases, respectively. Here, the bin width τ and number of bins is *N* are 10 ps and 1021, respectively. We can observe the near-uniform JTI with low coincidence counts. For Fig. 6, the duration of measured coincidence counting is 3 s, and no subtraction of background or accidental counts is performed.

We also quantify how close are the time-frequency bases to be mutually unbiased by evaluating Δ*M*, the normalized Frobenius norm $\frac{1}{2}{\left\|\cdot \right\|}_{F}$ of the difference between the normalized time-frequency bases’ correlation matrix *C*_TF_ and the ideal correlation matrix for MUBs $C_{\text{MUBs}}=\frac{1}{d^{2}}1_{d\times d}$ with ${\left(1_{d\times d}\right)}_{\mathrm{ij}}=1$ for all i,j∈{1,…,d}

$$\Delta M≔\frac{1}{2}{\left\|C_{\text{TF}}-C_{\text{MUBs}}\right\|}_{F}=\frac{1}{2}\sqrt{\sum_{i,j=1}^{d}{\left|{\left(C_{\text{TF}}\right)}_{\mathrm{ij}}-\frac{1}{d^{2}}\right|}^{2}}$$ (19)

The calculated Δ*M* values for different local dimensions *d*, in which we certify entanglement and evaluate secure key rates, are shown in Table 3.

**Table 3.** Normalized Frobenius norm of the difference between the normalized time-frequency bases’ correlation matrix *C*_TF_ and the ideal correlation matrix for MUBs *C*_MUBs_. This measure of the basis biasness is denoted by Δ*M* and is shown here (rounded to 1 significant figure) for various local dimensions *d*.

*d*	3	7	13	31	61	127	251	331	419	509	1021
Δ*M*	0.03	0.01	0.007	0.003	0.002	0.0008	0.0006	0.0007	0.0005	0.0005	0.0005
